# Manipulation of macrophage signaling by *Leishmania* virulence factors

**DOI:** 10.1080/21505594.2025.2549802

**Published:** 2025-09-08

**Authors:** Dhiraj Gurjar, Neelam Bodhale, Divanshu Shukla, Debadatta Nayak, Nibedita Lenka, Bhaskar Saha

**Affiliations:** a Division of Stem Cells and Immunobiology, National Centre for Cell Science, Pune, India; b Duncan Cancer Center, Baylor College of Medicine, Houston, TX, USA; c Department of Microbiology, University of Pennsylvania, Philadelphia, PA, USA; d Homeopathic Research Division, Central Council for Research in Homeopathy, Janakpuri, New Delhi, India; e Clinical HIV Laboratory, JSPS Government Homeopathic Medical College, Hyderabad, Telangana, India

**Keywords:** *Leishmania*, pathogenicity, virulence factors, signalling pathways, vaccine

## Abstract

*Leishmania*, a macrophage-residing parasite, expresses virulence factors that intercept macrophage signaling and inflicts leishmaniasis. Recently described virulence factors- eEF-1α (eukaryotic elongation factor), LmjF_36_3850 (*Leishmania major* F_36_3850), LdTyrPIP_22 (LDBPK_220120.1) and LmjMAPK (*L. major* mitogen activated protein kinase)-4/12 selectively modulate the activities of kinases, phosphatases and metabolism of phosphatidylinositol influencing the infection outcome. LmjF_36_3850, abundant in virulent *L. major*, interferes with PKC (Protein kinase C) activation; OAG (1-oleoyl-2-acetyl-sn-glycerol) supplementation enhanced PKC phosphorylation, increasing IL-12, but reducing IL-10, production and increased disease-promoting T cells. LdTyrPIP_22, a dual-specificity phosphatase, dephosphorylates phosphotyrosine residues and PI(3)P/PI(4)P, within the flagellar pocket and vesicles, suggesting a role in phosphoinositide (PI) signaling during differentiation. Its *L. mexicana* ortholog, LmDUSP1 (Dual-specificity Phosphatase), is a virulence factor linked to infectivity. 170 PX-domain-containing proteins in Kinetoplastea are implicated in phosphoinositide-mediated signaling, transport, and membrane trafficking. This review constructs a new framework of virulence factor-modulated host cell signaling as a bi-directional host–parasite interaction.

## Introduction

Leishmaniasis is a phlebotomine sandfly-transmitted parasitic disease, caused by the infection with more than 20 distinct *Leishmania spp*. In the gut of sandfly, *Leishmania* exists as flagellated, motile, extracellular promastigotes that, when transmitted to a mammal, are transformed into an aflagellate, sessile, intracellular amastigotes within the phagocytic cells such as macrophages [1]. According to the most recent WHO data, leishmaniasis is the second most deadly neglected tropical disease with approximately 0.7–1.0 million new cases per year and more than 150 million people worldwide are at risk of infection living in the tropical and subtropical areas [2]. Leishmaniasis shows a wide spectrum of clinical manifestations, including cutaneous (CL), muco-cutaneous (MCL), and visceral (VL) or kala-azar [3].

**Figure 1.**
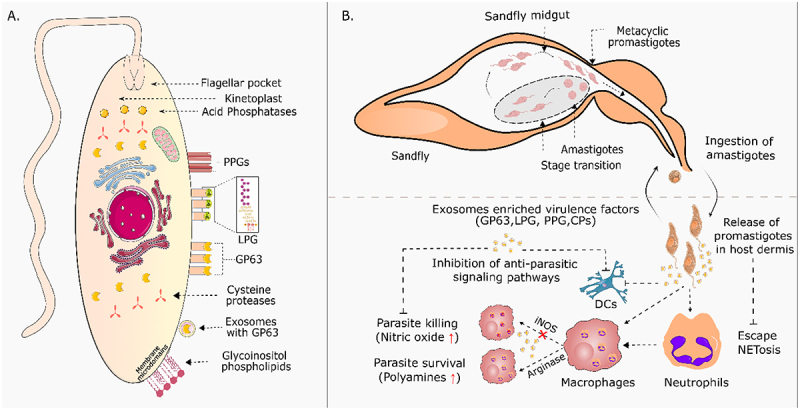
[Left] the classically known leishmania virulence factors and their localization within promastigotes. [right] schematic representation of leishmania transmission and survival mechanisms. Sandflies inject leishmania into the host, where they encounter the host immune pathways. With the aid of virulence factors, these parasites establish and sustain their survival within host macrophages.

The pathogenesis of leishmaniasis is regulated by multiple factors including the *Leishmania* strain/species, its virulence factors, and host genetics. *Leishmania* and other intracellular pathogens ensure their replication and persistence within host cells by expressing the virulence factors [Figure 1(A)] that intercept a complex network of proteins and cellular processes known as signal transduction.

In *Leishmania*, virulence factors such as lipophosphoglycan (LPG), 63kDa glycoprotein (GP63), phosphatases, and secreted exosomes enhance the longevity of infected host cells by mediating macrophage attachment and entry, resisting oxidative stress, supporting energy production, and modulating host signaling pathways to suppress pro-inflammatory responses and promote parasite survival [4]. However, *Leishmania* virulence is not absolute but relative, varying based on parasite strain, host immune response, and environmental conditions. These virulence factors, gene expression variability, and environmental conditions significantly influence how virulent a strain can be. Different *Leishmania* species and strains possess unique virulence factors that help them establish infection. Therefore, these different strains exhibit varying degrees of pathogenicity based on their interactions with host immune responses. For instance, virulence is influenced by a parasite’s ability to manipulate host immune responses, with highly virulent strains often inducing immunosuppression to thrive within macrophages or promoting a Th2-skewed response that favors persistence and disease progression, while less virulent strains may trigger a stronger immune response [5]. In addition to parasite-derived virulence factors, *Leishmania* also exploits host-derived molecules, which, when manipulated to aid parasite survival and immune suppression, function as proxy virulence factors - a concept reflecting their indirect but critical role in pathogenesis through pathogen-directed host regulation. Therefore, understanding both the molecular mechanisms of *Leishmania* virulence and the host’s immune landscape can result in more effective treatment strategies and anti-leishmanial vaccine development.

These virulence factors also represent novel therapeutic and prophylactic targets. Novel therapeutic targets are required, as increasing emergence of drug-resistance that limits the efficacy of the treatment in leishmaniasis becomes a global health concern. The last seven decades of search for a *Leishmania* vaccine has yielded only failures. Herein, we provide an overview of recent discoveries in which *Leishmania* virulence factors manipulate the host regulatory molecules and signaling to prevent the macrophages activation. We mechanistically integrate these virulence factors in the framework of *Leishmania*-host interactions for understanding their roles in establishing the infection and how their targeting may lead to therapy or prophylaxis.

## The leishmania secretome

*Leishmania*, despite lacking classical secretion systems (Type I – VII), has evolved specialized mechanisms to export virulence factors and modulate host interactions. Exosome-mediated secretion, functionally analogous to Type I and Type IV secretion systems, allows the parasite to deliver effector proteins and RNA molecules that influence the host immune response [6,7]. The flagellar pocket, a crucial hub for protein trafficking, operates similarly to Type III and Type VI secretion systems by facilitating the controlled release of virulence factors and nutrient uptake [8]. *Leishmania* employs unconventional shedding mechanisms, reminiscent of Type V secretion, to release surface-bound proteins such as GP63, aiding in host cell invasion and immune evasion [9]. Furthermore, by manipulating lysosomal pathways – similar to Type VII secretion - *Leishmania* alters host cell signaling and survival, ensuring its persistence within macrophages [10]. Among these secretion mechanisms, *Leishmania*, like other higher eukaryotes, preferably uses an ER-Golgi secretion pathway, with secretion directed through the flagellar pocket [11]. The parasite flagellum initiates the contact between the promastigote and the host macrophage which, in turn, results in the release of certain factors by the parasites causing modulation of macrophage phagocytic activity [Figure 1(B)] [12]. A recent study revealed that small extracellular vesicles of *Leishmania* contain specific protein signatures reflecting the drug-resistance profile of their parental *Leishmania* strain [13]. These vesicles are enriched with drug resistance genes and virulence factors, and they are capable of transferring these elements to boost the growth and fitness of naïve recipient parasites [14]. *Leishmania* exosomes enhance virulence by delivering RNA cargo that modulates host immune responses and promotes infection. RNAi-proficient *L. braziliensis* uses siRNA-containing exosomes to silence target genes, a mechanism absent in RNAi-deficient *L. donovani* [15]. Additionally, *Leishmania* extracellular vesicles can act as a viral envelope for *Leishmania* RNA virus 1 [LRV1], boosting its transmission and infectivity [16]. LRVs are significant because LRV2^+^ in *L. major* enhances the expression of virulence factors (GP63, HSP83, hsp70, cpb, and MPI) and reduces proinflammatory biomarkers (NLRP3, IL-18, IL-12, and IL-1β), potentially altering disease outcomes and enhancing *Leishmania* survival [17,18]. Silverman et al. suggest that *L. donovani* exosomes could fuse within macrophage phagolysosomes, releasing their contents into the infected cells cytoplasm [19]. These factors include lipophosphoglycan (LPG), GP63, proteophosphoglycans (PPG), etc., which are considered as the essential determinants of parasitic virulence and needed for invasion of immune system [Figure 1, Right] [7,20,21]. These studies reported that *L. donovani* exosomes inhibited IFN-γ-induced IL-8 and TNF-α while promoting IL-10 production in human monocytes when co-administered with parasites. They also reduce CD80 and HLA-DR expression in matured monocyte-derived DCs. The promastigotes are taken up and internalized in the phagosomes by macrophages. Later, phagosomes fuse with lysosome forming phagolysosomes with acidic environment, free radicals of nitrogen and oxygen, and various antimicrobial enzymes but parasites survive in this challenging and hostile environment [22] highlighting the *Leishmania* exosomes’ functions in regulating immune responses and pathogenesis.

## Leishmania virulence factors interfere with various signaling events

Herein, we discuss the major virulence factors employed by *Leishmania* parasites, elucidating their significant roles in altering various signaling pathways within the host.

### GP63

**Figure 2.**
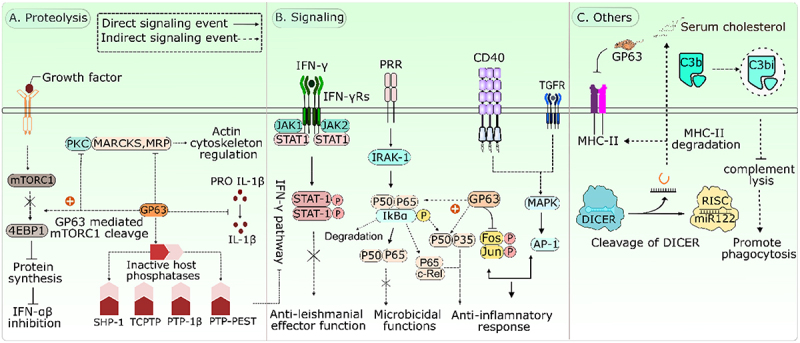
Leishmania metalloprotease GP63 utilizes three distinct strategies to impair anti-leishmanial immune responses: (A) proteolysis, (B) disruption of signaling pathways, and (C) inhibition of complement lysis. (A) GP63 cleaves several host proteins, including mTORC1, MARCKS, MRP, and phosphatases, disrupting host cell signaling. This affects actin cytoskeleton regulation, IL-1β maturation, and suppresses protein synthesis via mTORC1/4EBP1 inhibition. (B) leishmania disrupts key host signaling pathways to evade immune responses. It blocks IFN-γR – JAK1/2–STAT1 signaling, impairing macrophage killing. PRRs pathways are modulated via IRAK-1 and cleavage of p65 (RelA) into p35 and p50, reducing NF-κB activation. GP63 also cleaves c-jun and c-Fos, disrupting AP-1 activity and suppressing inflammatory responses. (C) GP63 impairs MHC-II expression and cleaves DICER, disrupting miRNA (e.g. miR-122) processing to aid immune evasion. It also converts C3b to C3bi, avoiding complement lysis while enhancing phagocytosis.

The zinc-dependent metalloprotease, glycoprotein 63 or leishmanolysin, a major surface glycoprotein on *Leishmania*, significantly impacts host cell signaling mechanisms and associated functions, has been extensively studied owing to its abundance and role in virulence. GP63 is abundant in promastigotes but has been demonstrated to be downregulated in amastigotes, suggesting it plays different roles in promastigotes and amastigotes [23]. GP63 has been shown to subvert the host complement system, to prevent complement-mediated lysis. Studies have shown that *Leishmania* overexpressing the proteolytically active GP63 is able to convert C3b into their inactive form, iC3b, thereby disrupting the complement cascade and increasing resistance to complement-mediated lysis [24]. Later, iC3b is recognized by macrophages surface receptors, which promotes phagocytosis without formation of the membrane attack complex. GP63 promotes iC3b-/C3b-opsonized parasite uptake through CR1 receptor resulting in reduced respiratory burst impairing *Leishmania* killing [Figure 2] [25–27].

GP63 has been found to impact multiple-signaling pathways in macrophages associated with microbicidal functions. Myristoylated-Alanine Rich C-Kinase Substrate and MARCKS Related Protein, involved in regulation of cytoskeletal actin, are substrates for GP63. GP63 inhibitors prevented MRP hydrolysis by GP63 during *L. major* infection; similar outcome emerges when GP63 proteolytic site on MRP is mutated [Figure 2] [28]. GP63 has been implicated in modulating the IFN-γ signaling pathway in macrophages during leishmaniasis. It was shown that GP63 was responsible for activation for host cell PTPs, negative regulatory molecules, known to greatly influence the IFN-γ pathway in *Leishmania*-infected macrophages. Research employing *L. major* wild-type and GP63-deficient (GP63^−/−^) strains has conclusively demonstrated that GP63 is responsible for the activation of host-derived proxy virulence factors – such as SHP-1, TC-PTP, PTP1B, and PTP-PEST – through the cleavage of their C-terminal domains [Figure 2] [29]. GP63 is shown to attenuate PKC activity via their proteolytic effect and diminish the release of ROS [30]. GP63 degrades c-Jun, disrupting AP-1 function and reducing the expression of anti-parasitic genes, pro-leishmanial cytokines, and nitric oxide in macrophages [Figure 2] [31]. The mTOR pathway tightly regulates 4E-BP1, but in leishmaniasis, GP63-induced cleavage of mTOR blocks mTORC1 activation and inhibits 4E-BP1 phosphorylation. When 4E-BP1 is not phosphorylated, it binds to eIF4E, preventing eIF4E-eIF4G interaction and disrupting global protein translation in macrophages [32]. GP63-deficient parasites are unable to suppress global protein translation in these infected cells. Additionally, GP63 has been reported to modulate NF-κB signaling by targeting distinct subunits, including p65 (RelA) and c-Rel-two functionally important NF-κB family members involved in transcriptional regulation of inflammatory responses. GP63-mediated cleavage of p65 (RelA) generates a truncated p35 (RelA) fragment that translocates to the nucleus, forms heterodimers with NF-κB p50, and binds DNA with altered specificity [Figure 2] [33]. c-Rel has also been implicated in *Leishmania*-induced immune modulation. It has been demonstrated that *L. major* infection inhibits PMA-induced nuclear translocation of the canonical p50/p65 complex in differentiated U937 cells, while inducing transcriptionally active p50/c-Rel complexes in both U937 cells and primary human monocytes. While the direct involvement of GP63 in c-Rel modulation remains to be fully defined, these c-Rel – containing heterodimers are associated with increased IL-10 production, contributing to an anti-inflammatory environment that favors parasite survival [34]. Furthermore, *L. donovani* exploits GP63 to suppress Dicer, leading to the downregulation of miR-1914-3p and overexpression of Rab18 and TRAPPC9. This drives lipid body biogenesis and their recruitment to parasitophorous vacuoles, supplying fatty acids essential for parasite growth [35]. Evidence indicate that GP63 influences the antimicrobial functions of macrophages, emphasizing its pivotal role as a virulence factor that contributes to the parasite’s survival and holds significant therapeutic potential [36].

### Lipophosphoglycan [LPG]

**Figure 3.**
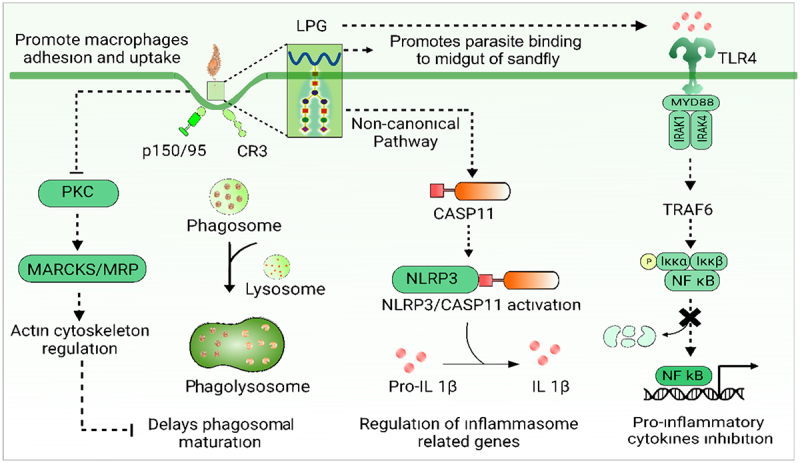
Leishmania LPG alters macrophage signalling for immune Evasion. LPG-induced modifications in macrophage promotes macrophage adhesion via CR3 and p150/95, delays phagolysosomal maturation, suppresses pro-inflammatory cytokines through TLR4-MyD88-NF-κB signaling, and activates CASP11-NLRP3 inflammasome. It also impairs PKC activation and ROS production, facilitating immune evasion and infection survival.

LPG is the most abundant glycoconjugate on the surface of *Leishmania* parasites, and plays a crucial role as virulence factor in the ability to evade the host immune system and establish infection. The side-chain composition of LPG varies in length during different stages of *Leishmania* forms, Procyclic promastigotes have shorter length, and metacyclic amastigotes have higher length, whereas it is undetectably low in amastigotes. This indicates that LPG likely plays a role in parasite entry and early infection stages. The entry process is facilitated by the interaction between Promastigote LPS and the CR3 and the integrin receptor p150/95 [Figure 3] [37]. It is shown that LPG-deficient avirulent *L. major* is rapidly eliminated within 18 hours. However, when LPG is transferred by a virulent strain, it enables the promastigotes to survive inside host macrophages [38]. Synthetic *Leishmania* LPGs act by stimulating ERK-1/2 to inhibit macrophage IL-12 production [39]. LPG treatment of *L. mexicana*-infected BMDMs results in suppressed IL-12 production due to the interaction between TLR4 and LPS [40]. Macrophages from the LPS-resistant SPRET/Ei mouse strain maintain normal MyD88-mediated signaling [Figure 3]. They display a defect in the production of type-1 interferons and poor induction of IFNβ-dependent genes [40]. Studies have shown LPG-mediated modulation of various signaling pathways via inhibition of PKC [Figure 3] [41,42]. IL-1β and TNF-α expression is reduced when THP-1 cells are treated with LPG [43]. LPS or DAG treatment of *L. donovani*-infected macrophages results in the inhibition of PKC-dependent c-Fos expression [44]. LPG reduces iNOS expression in macrophages and inhibits the production of macrophage IL-12, both are critical for control of leishmaniasis [45,46]. Studies identify that LPG is responsible for CASP11 activation and also initiates the noncanonical activation of NLRP3. When LPG is introduced into macrophages, it leads to CASP11 activation, and infections carried out using Lpg1^−^**/**^−^ parasites result in reduced CASP11/NLRP3 activation [Figure 3] [47]. LPG impaired the proper activation of PKCα and PKCβ and inhibit ROS production [48,49].

### Elongation factor-1α

**Figure 4.**
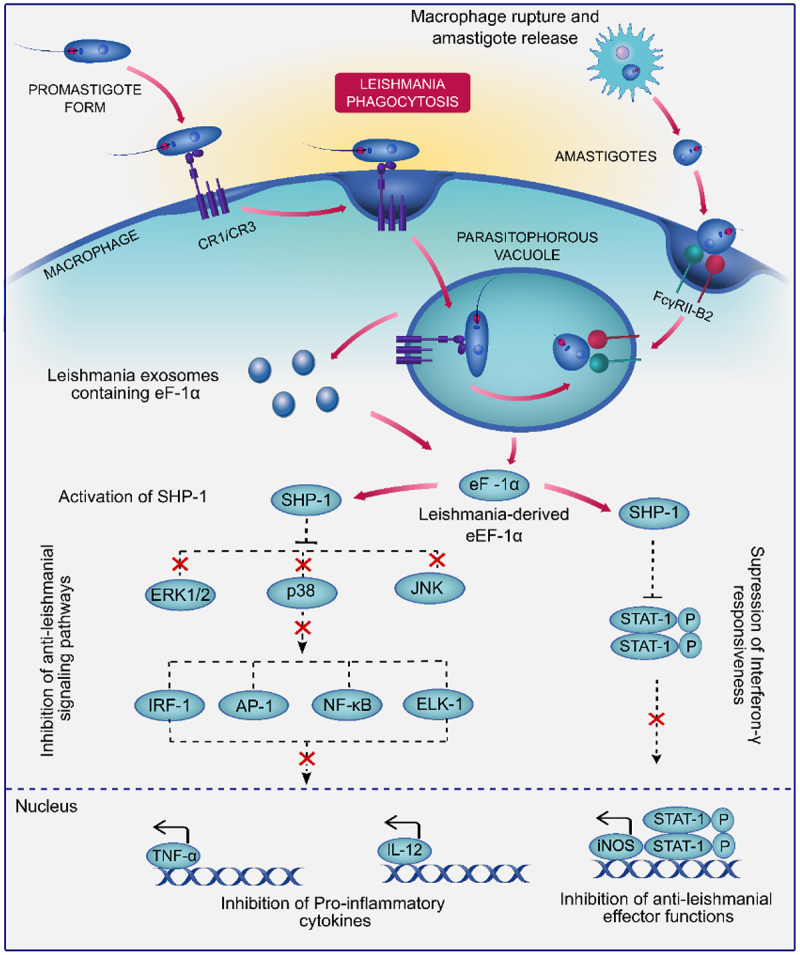
Mechanism of leishmania eEF-1α–mediated immune evasion in macrophages. Leishmania eEF-1α mediates immune evasion by activating host SHP-1 phosphatase, which inhibits MAPK (ERK1/2, p38, JNK) and STAT1 signaling. This suppresses pro-inflammatory cytokines (TNF-α, IL-12) and iNOS expression, impairing anti-leishmanial responses and promoting parasite survival in macrophages. (elements from reactome, CC by 4.0.).

EF1α is a key enzyme in protein synthesis, facilitating the GTP-dependent binding of aminoacyl-tRNA to ribosomes and captures deacylated tRNA during translation. Moreover, eEF1 acts as a key hub in protein networks, linking hundreds of partners [50]. In *Leishmania*, eEF1α has been identified within exosomes, where it plays a crucial role in immunosuppression. It binds and activates SHP-1 both *in vitro* and *in vivo*, and block the induction of iNOS in response to IFN-γ, leading to deactivation of macrophages during leishmaniasis [Figure 4] [19,51]. The proteome analysis of *L. infantum* demonstrates that eukaryotic eEF1α is up-regulated in the stationary phase of promastigote cultures at both protein and mRNA levels, indicating its critical role in the parasite’s life cycle. The study also identifies a low-molecular-weight isoform and an alternatively processed form of eEF1α, suggesting its functional diversity [52]. In a study involving BALB/c mice, the in vivo administration of truncated and liposomal EF1-α was observed to trigger a Th1 immune response, marked by elevated levels of IFN-γ, IL-12, and TNF-α with concurrent suppression of IL-4, IL-10, and TGF-β levels, ensuring protective response in leishmaniasis [53]. A recent study has identified EF-1α as the only protein in *L. infantum* promastigotes with a phosphorylcholine (PC) modification. The researchers use 2D-gel proteomics and Western blot analysis with the PC-specific antibody TEPC-15 to examine eEF1α. This modification is crucial for the interaction between *L. infantum* EF-1α and the host protein SHP-1, which plays a significant role in the parasite’s virulence and ability to evade the host immune response [54]. Another elongation factor eEF1β, tested against *L. infantum*, induced a Th1 immune response and reduced parasite burden in mice. The recombinant protein plus adjuvant provided better protection than the DNA vaccine, showing its potential as an effective vaccine candidate [55]. A similar study was performed using eEF-2 from *L. donovani* to evaluate its potential as a vaccine for visceral leishmaniasis. Recombinant LelF-2 induced a robust Th1 immune response, enhancing the production of IFN-γ, IL-12, and TNF-α, while suppressing IL-4 and IL-10. This response led to significant protection against *L. donovani* infection, positioning LelF-2 as a promising candidate for further development in VL vaccine strategies [56]. A study identified *L. major* eEF1B, which, when reconstituted, exhibits trypanothione S-transferase and peroxidase activities, particularly against linoleic acid hydroperoxide. *L. major* eEF1B also plays a role in protecting the parasite from lipid peroxidation. Immunofluorescence suggests its localization to the endoplasmic reticulum, linking it to both protein synthesis and oxidative stress defense [57].

### Phosphatases

Cell signaling is primarily controlled by two key classes of enzymes: protein kinases and phosphoprotein phosphatases. Signal transmission involves kinase and phosphatases cascades with phosphorylation and dephosphorylation, which parasites often disrupt. Therefore, phosphatases can contribute to pathogenesis by disrupting host signaling, aiding nutrient acquisition, and altering immune responses [58].

In *L. donovani*-infected macrophages, the host production of ceramide following the uptake of the parasite triggers the activation of PP2A. Consequently, AKT undergoes dephosphorylation and reduced TNF-α secretion [59]. PP2A activation is responsible for the unresponsiveness of mouse lymphocytes to strong stimuli like PMA and ionomycin, but this effect can be reversed by treating lymphocytes with okadaic acid, a PP2A-specific inhibitor [60]. Studies indicate that the activation of both PP1 and PP2A can impede the production of iNOS and subsequent NO production negatively impacting anti-leishmanial defense [61]. PKC-ζ is responsible for the induction of PP2A and MKP3 which plays a significant role in the inhibition of iNOS expression through ERK1/2 deactivation. Cystatin inhibits the MKP3 (DUSP6) and PP2A activity, which favors the induction of TNF-α, iNOS and T cell response and reduce parasite burden [62]. In the same study, PKC-ε activation led to the activation of MKP-1 (DUSP1) that, in turn, inhibited p38MAPK. *L. major*-infected anti-CD40-treated or DUSP6-silenced macrophages show activation of ERK, an increased production of IL-10, and a decrease in the expression of IL-12 and iNOS. Conversely, when DUSP1 was inhibited, it resulted in the activation of p38 and an elevated production of IL-12 and iNOS [63]. To study the functional reciprocity of DUSP1 and DUSP6, a coinfection of macrophages with *Mycobacterium indicus pranii* and *L. donovani* results in increased MKP-3 but decreased MKP-1 expression. Following *M. pranii* infection, there is an elevated association between PKC-β and DUSP6, which leads to a reduction in IL-10, decreased phosphorylation of ERK1/2, and lower expression of Arginase-1. It reduces the interaction between PKC-ε and DUSP1, resulting in an increase in IL-12 production, phosphorylation of p38 and iNOS expression. Therefore, silencing DUSP1 or boosting DUSP6 in BALB/c mice infected with *L. donovani* reduces parasite load by increasing IL-12 and decreasing IL-10 levels [64]. Another dual-specificity phosphatase, DUSP4, shares functions similar to DUSP6. In *L. mexicana*-infected DUSP4^−/−^ mice, JNK and p38 phosphorylation and Arg1 expression increase in macrophages favoring Th2 response [65].

In *L. donovani*-infected macrophages, SHP-1 (PTPN6) becomes rapidly activated and suppresses IFN-γ signaling by interacting with JAK2 and STAT1 [66]. SHP-1 also downregulates MAPK and transcription factors like c-FOS, NF-κB, ERK1/2 and AP-1, thereby inhibiting iNOS and NO production [67,68]. In *L. donovani* infection, fructose 1,6-bisphosphate aldolase is secreted and can activate SHP-1 phosphatase activity in vitro [69]. *Leishmania* infection triggers rapid SHP-1 activation, which binds to and inhibits IRAK-1. This prevents IRAK-1 detachment from TLR4 adaptor MyD88 and attach to TRAF6, impairing macrophage responses to LPS [70]. *L. major* binding to the Mincle receptor impairs dendritic cell activation by recruiting SHP-1, which converts the receptor’s ITAM in the FcRγ-chain signaling into an inhibitory motif (ITAMi) [71]. In macrophages, Siglec-1 helps uptake *L. donovani* parasites, while Siglec-5 binding reduces ROS and NO production, promoting a Th2 response due to SHP-1 recruitment and immune cell deactivation [72].

**Figure 5.**
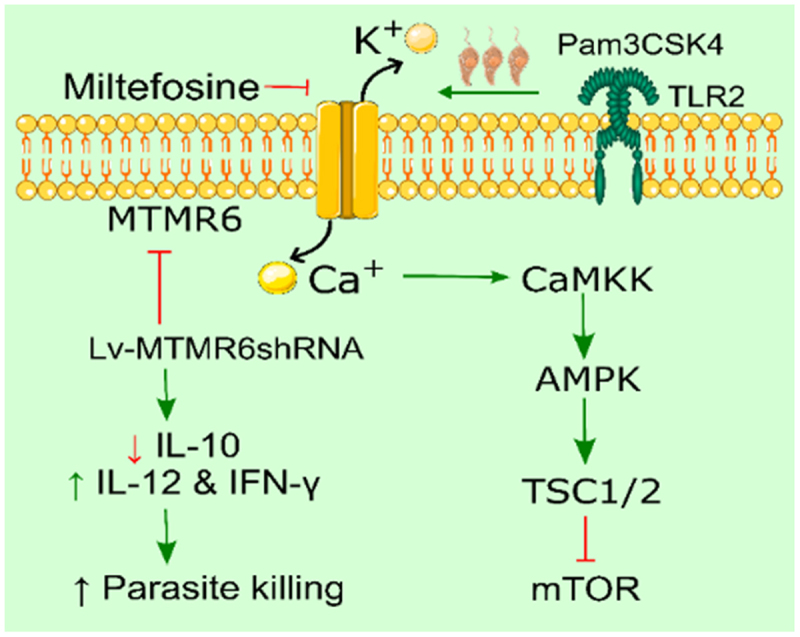
Leishmania infection upregulates MTMR6, an ion channel-associated phosphatase. Silencing MTMR6 reduces parasite growth, infection, and IL-10 while enhancing IL-12 and IFN-γ immunity. MTMR6 is induced in macrophages via TLR1/TLR2 signaling and downregulated by miltefosine. It may impair T cell function by modulating membrane potential and Ca^2 +^ influx.

To identify the phosphatases that regulate *Leishmania* infection, we conducted a screening of a human phosphatase siRNA library for anti-leishmanial functions in THP-1 macrophage-like cells. We identified seven phosphatases that influenced leishmanicidal activity and cytokine (IL-10 and IL-12) production, initially in THP-1 cells and later in monocyte-derived macrophages from healthy volunteers and VL patients before and after miltefosine treatment. Through this screening, we identified MTMR6, an ion channel – associated phosphatase, as a proxy virulence factor that suppresses anti-leishmanial immunity by modulating macrophage and T cell responses. MTMR6 may negatively regulate T cell functions by influencing cellular depolarization, membrane potential, Ca^2 +^ influx, and phosphatidylinositol phosphate availability. In leishmaniasis, this regulation could contribute to T cell suppression, either by blocking the second signal needed for activation or through negative signaling via PD-L1 and CTLA4, leading to hypo-responsiveness [73]. Silencing MTMR6 with Lv-MTMR6shRNA reduced *Leishmania* growth, infection in BALB/c mice, amastigote counts, IL-10, and enhanced IL-12 and IFN-γ-mediated immunity. Virulent *L. donovani* infection increased MTMR6 expression in macrophages, which was enhanced by the TLR1/TLR2 ligand Pam3CSK4 and reduced by TLR2 blockade. Miltefosine treatment decreased MTMR6 expression in both infected macrophages and VL patients [Figure 5]. MTMR6 emerges as a TLR2-modulated ion channel-associated phosphatase, crucial in VL pathogenesis and anti-leishmanial immunity, making it a promising therapeutic target against leishmaniasis [74,75].

Another study from our group shows that 3-alpha-amino-cholestane (3AC) selectively inhibits SHIP1, an inositol-5’-phosphate-specific phosphatase crucial for immune regulation. We found that SHIP1 expression increases in *Leishmania*-infected macrophages following treatment with 3AC. In *Leishmania*-susceptible BALB/c mice, 3AC enhances anti-leishmanial cytokines, reduces pro-leishmanial cytokines, and decreases parasite load in *L. major* and *L. donovani* infections [76]. Altogether, these findings emphasize the central role of phosphatases in modulating host responses and their promise as therapeutic targets in leishmaniasis.

***Kinases:*** Kinases in *leishmania* are critical for regulating essential processes such as cell cycle progression, differentiation, immune evasion, and metabolism, which are crucial for the parasite’s survival and virulence. Protein Kinase A (PKA), Cyclin-Dependent Kinases (CDKs), and MAP Kinases (MAPKs) control differentiation, cell division, and stress responses. Protein Kinase C (PKC) regulates adhesion and motility, while *Leishmania*-specific kinases modify surface molecules for immune evasion. Tyrosine kinases influence host–parasite interactions, AMPK manages energy metabolism, and PI3K governs growth and survival [77].

The interactions between MAPK and ERK in *Leishmania* infections determine whether the immune response clears the parasite or allows it to persist, leading to chronic disease [78]. Previously, we demonstrated the expression profiles of 15 MAPKs in different strains of *L. donovani*, including both virulent and avirulent forms, revealing distinct differences in their expression patterns [79].

Among the 15 MAPKs, LmjMAPK4 was more highly expressed in virulent *L. major* and was localized in both parasitophorous vacuoles and the cytoplasm. LmjMAPK4 binds MEK-1/2 but not MKK-3/6, and overexpression enhanced CD40-activated MEK-1/2-ERK-1/2-MKP-1 signaling while inhibiting MKK3/6-p38MAPK-MKP-3. Inhibition of LmjMAPK4 restored CD40-triggered host-protective anti-leishmanial functions in BALB/c mice [80]. In contrast, *L. donovani* infection can be targeted through LmjMAPK10 DNA vaccination, which significantly reduces parasite burden and enhances T-cell responses, TH1-type cytokines, and IgG2a production. This protection correlates with increased IL-12 and IFN-γ levels, and decreased IL-10 and IL-4, pointing to a strong immune response [81]. Co-treatment with MAPK10 and IL-7 offers stronger protection, reducing splenic and hepatic parasite loads more effectively than MAPK10 alone, suggesting a robust memory T-cell response [82]. A recent study identifies LdMAPK12 as a potential virulence factor in *L. donovani*, present in both promastigotes and amastigotes, with higher expression in virulent promastigotes. Cytokine stimulation in macrophages alters LdMAPK12 expression, with pro-inflammatory cytokines reducing and anti-inflammatory cytokines increasing its expression [83].

A recent study from our group has shown that *L. donovani* LPG regulates TLR2 signaling via TPL2, a MAPK module component, in macrophages. Blockade of TLR2 or TPL2 altered p38MAPK and ERK-1/2 activation, with TLR2 blockade inhibiting TPL2 activation. In *L. donovani* infection, LPG inhibited Akt activation, and TPL2 inhibition prevented this effect. TLR2 blockade reduced phosphorylation of p38, Akt, and TPL2, while ERK1/2 phosphorylation increased. TPL2 inhibition also reduced TGF-β expression, increased TNF-α, and decreased the amastigote counts. LPG selectively increased TLR2 expression and modulated p38MAPK and AKT signaling. Molecular docking indicated that the glycan-chain length of LPG affects its interaction with TLR2, suggesting TPL2 as a key virulence factor in *L. donovani* pathogenesis [84,85]. We have demonstrated that *L. major* infection increases TLR11/12 expression, which is augmented by LPG-TLR2 interaction. Blocking either LPG or TLR2 prevents this heightened TLR11/TLR12 expression. LPG-TLR2 interaction activates MyD88- and TIRAP-mediated signaling, leading to enhanced ERK1/2 activation and increased IL-10 production, which in turn promotes TLR11/TLR12 expression. Silencing TLR11/12 reduces parasite burden, increases IFN-γ levels, and decreases IL-4 production, indicating a pro-leishmanial role for TLR11/12 [86]. Studying TLR effects on CD40 expression, we found that TLR-3 (poly I:C), TLR-4 (LPS), TLR-7/8 (imiquimod), and TLR-9 (CpG) ligands upregulate CD40, while CD40 stimulation selectively enhances TLR-9 expression. Upregulated CD40 enhances macrophage activation, cytokine production, and anti-*Leishmania* immunity [87]. We also demonstrate that *L. major* lipophosphoglycan suppresses host-protective responses by downregulating TLR-9 expression via TLR-2 activation. Infection with *L. major* expressing high LPG levels decreases TLR-9 expression, which is reversed by anti-LPG and anti-TLR-2 antibodies. LPG-mediated suppression of TLR-9 is driven by TGF-β and IL-10. Blocking LPG or TLR-2 enhances parasite clearance in macrophages, while co-treatment with anti-TLR-2 and CpG in mice reduces footpad swelling, parasite load, and promotes an IFN-γ-driven immune response [88]. These findings explain how *Leishmania* exploits inter-TLR interdependency to manipulate TLR signaling crosstalk and evade host immunity, uncovering potential therapeutic targets for leishmaniasis intervention [89].

### Cysteine proteases

Cysteine proteases, also known as thiol proteases, are vital virulence factors in *Leishmania*, aiding in tissue invasion, immune evasion, and inflammation, and are key players in leishmaniasis pathogenesis. *L. mexicana* possesses three CP genes- cpa, cpb, and cpc- encoding papain-like proteases in Clan CA, Family C1, expressed in both promastigote and amastigote forms. In C3H mice, the *L. mexicana* ∆cpb deletion mutant does not induce lesions and is associated with reduced parasite burden, accompanied by a shift in the immune response toward Th1 [90]. At higher concentrations of *L. mexicana*, CD25 expression on T cells can be cleaved by CPB2.8 and dust mite Derp1, a part of the IL-2 receptor, which potentially reduce the T cell proliferation [91]. A purified cysteine protease (CPB) derived from *L. chagasi*, as well as *Leishmania* whole-cell lysates, has the capability to activate human TGF-β [92]. Another study identifies that *Porphyromonas gingivalis* cysteine proteinases can hydrolyze IL-12, reducing the IL-12 induced IFN-γ production from CD4^+^ T cells [93]. There are reports suggesting that CPB facilitates the degradation of NF-κB and IκB, leading to a suppression of IL-12 production [94]. As these proteases are key in various processes, developing effective protease inhibitors is crucial for advancing therapeutic options in leishmaniasis.

### 11-kDa kinetoplast membrane protein [KMP-11]

KMP-11 is an 11-kDa protein found in kinetoplast parasites, including *Leishmania*. It is a highly conserved surface protein found in both promastigotes and amastigotes, and their expression is more in metacyclic promastigotes. Recently, a study identifies CHOL interaction motifs in KMP-11, including two CRAC-like and one CARC domain, conserved across *Leishmania* species. It also reveals KMP-11 structural similarity to lipid transfer proteins, suggesting its role in lipid-mediated host cell invasion. This study demonstrated that the *L. donovani* protein KMP-11 plays a crucial role in host cell invasion by forming oligomers that bridge LD and macrophages membranes. This interaction is influenced by differences in cholesterol and ergosterol levels in their membranes, which are essential for key invasion steps: (a) initial attachment, (b) CHOL transport from macrophages to LD, and (c) LD detachment via a liquid-ordered to liquid-disordered membrane-phase transition. KMP-11 depletion impairs LD attachment and invasion, highlighting its importance. A mathematical model, developed through tryptophan-scanning mutagenesis and synthesized peptides, reveals that the hydrophobic moment and symmetry sequence code of KMP-11 membrane-interacting domain drive membrane-phase transitions, facilitating infection. The transition is linked to lipid raft disruption and T-cell deactivation, aiding parasite internalization [95]. Another study explores KMP-11 interaction with phospholipid membranes and cholesterol influence. Using biophysical techniques, they determined binding constants for wild-type (WT) KMP-11 and single-site tryptophan mutants. In the absence of cholesterol, partially exposed (Y5W) and buried (Y48W) mutants showed higher membrane-binding affinities than WT. Cholesterol reduced binding for WT and Y48W but increased it for Y5W and F77W. These studies reveal how KMP-11 conformation and its interaction with membrane lipids, including cholesterol, influence *Leishmania* survival in host macrophages [96]. They also show that since KMP-11 is found on the parasite’s membrane surface, it strongly associates with anionic phospholipid membranes, as indicated by reduced ζ-potential and decreased fluorescence intensity of membrane-bound dye. Fluorescence leakage and microscopy confirm KMP-11-induced pore formation in anionic membranes, whereas neutral phospholipid vesicles do not exhibit pore formation. This pore formation is inhibited by cholesterol, suggesting its role in leishmaniasis [97].

Although the impact of KMP-11 on signaling pathways hasn’t been explored, its potential as a vaccine candidate, which is capable of stimulating innate and adaptive host immune responses, has been investigated. KMP-11 along with HASPB was evaluated in BALB/c mice against *L. major* challenge infection. It resulted in the elevated levels of IgG2a and IFN-γ and Th1 cell activation [98]. Plasmid-based KMP-11 DNA immunization of hamsters elicited a combined Th1/Th2 T-cell response which was marked by high levels of IFN-γ, TNF-α, IL-4, and IL-12, while IL-10 was notably absent [99]. Although various studies have demonstrated the potential of KMP-11 as a vaccine candidate, further exploration is needed to better understand the signaling aspects associated with KMP-11.

### miRNA role in leishmania virulence

In the recent year’s microRNAs, a group of endogenously expressed small noncoding RNAs, have emerged as regulator of post-transcriptional gene expression. The role of miRNAs at the host – pathogen interface has been increasingly recognized, with evidence showing that pathogens exploit the host miRNA machinery to mislead immune responses; thus, these host-derived miRNAs, when hijacked, can function as proxy virulence factors that facilitate immune evasion and pathogen survival [100]. By disrupting miRNA expression, *Leishmania* could act on many target genes simultaneously [101]. Interestingly, these parasitic virulence factors interfere with miRNA machinery, affecting the regulation of host mRNA expression. The virulence factors mediated interference and alteration in the macrophage’s miRNAs profiling affects inflammatory genes, immuno-metabolic gene expression, that could lead to changes in nitric oxide production, macrophages polarization, cytokine production, phagolysosome maturation, attenuation of various signaling pathways, T cell proliferation, which ultimately affects host cell fitness. Therefore, identifying virulence factors that affect miRNA expression in *Leishmania* parasites and infected cells is crucial for understanding *Leishmania* pathogenicity and the immune response.

**Figure 6.**
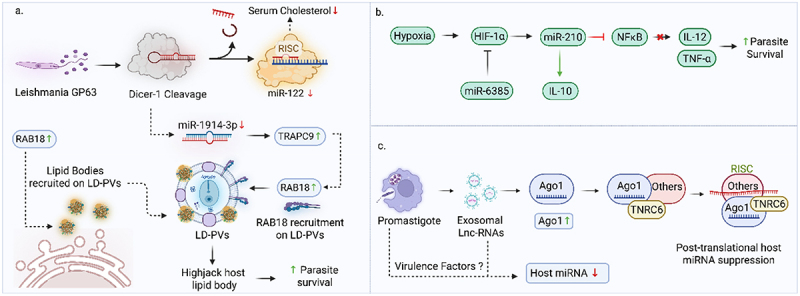
miRNA mediated interference in leishmania virulence. a) leishmania gp63 cleaves Dicer1, lowering miR-122 and serum cholesterol while promoting lipid biogenesis via miR-1914-3p suppression. b) HIF-1α-induced miR-210 suppresses NF-κB p50, inhibiting IL-12a and TNF-α activation in leishmania-infected macrophages. c) leishmania infection elevates host macrophage Ago1, activating RNAi complexes and promoting parasite survival via posttranslational host miRNA suppression.

The virulence factor glycoprotein gp63 in *L. donovani* targets the host Dicer1 enzyme, causing the cleavage of Dicer1. This action leads to the downregulation of pre-miR-122 and its subsequent processing into miR-122 [102]. Downregulation of miR-122 is coupled with low serum cholesterol in VL mice and conversely, restoring miR-122 or Dicer-1 in infected mouse liver resulted in high serum cholesterol and reduced liver parasite load [Figure 6(a)]. GP63 also suppresses miR-1914-3p, upregulating Rab18 and TRAPPC9 to drive lipid body biogenesis and fatty acid supply for parasite growth [Figure 6(a)] [34]. c-Myc has been identified as a proxy virulence factor of *Leishmania* that targets the miRNA machinery of the host. *Leishmania* infection significantly upregulates c-Myc, and silencing c-Myc counteracts this upregulation by reversing miRNA suppression. This significantly reduces *Leishmania* survival, highlighting c-Myc crucial role in the leishmaniasis [103]. Another study has shown that *Leishmania* infection induces a differential expression of 208 miRNAs in CD4^+^ T cells. The upregulation of miRNAs, targeting transcription factors that promote CD4+ T cell polarization to the Th1, associated with the IFN-γ signaling cascade, suggests that this miRNA increase could be a parasitic strategy to modulate IFN-γ pathway and enhance virulence [104]. Singh et al. demonstrated that miR-30a-3p is overexpressed in *Leishmania*-infected cells. Its downregulation in trans-dibenzalacetone-treated parasites suggests that reducing miR-30a-3p could decrease *Leishmania* infectivity and transmission [105]. In leishmaniasis, infected cells release extracellular vesicles containing virulence factors, with miR-146a, miR-21, miR-125b, and miR-16 at higher levels compared to EVs from control cells, enhancing infectivity [106]. During *Leishmania* infection, HIF-1α induces miR-210 levels in macrophages, which inhibits NF-κB-induced activation of IL-12a and TNF-α by affecting the p50 subunit [Figure 6(b)] [107]. Recently, a study identifies host macrophage Argonaute 1 (Ago1) as a proxy virulence factor in *L. donovani* pathogenesis. Infection increases Ago1 abundance and its incorporation into active RNAi complexes, crucial for parasite survival. Ago1 silencing significantly reduces *Leishmania* survival, while proteomic analysis shows Ago1 regulates key pathogenesis-related proteins [Figure 6(c)]. These findings position Ago1 as an essential virulence factor in *Leishmania* infection [108]. These information suggest the potential role that virulence factors may play in the alteration of miRNA, which in turn, could contribute to the development of leishmaniasis.

## Leishmania virulence and metabolism

In addition to these well-recognized virulence factors, *Leishmania* employs metabolic factors as integral components of its virulence strategy, thereby ensuring its prolonged survival, which in turn, contributes to the progression of the disease. The study of metabolic alterations in *Leishmania* species offer valuable insights and potential biomarkers for assessing virulence and monitoring the progression of the disease. Therefore, understanding how *Leishmania* virulence and metabolism are linked has significant preventive potential. The recent progress in proteomics and metabolomics has greatly enhanced our capacity to detect, identify, and quantify molecules. This breakthrough has the potential to provide deeper insights into the metabolic aspects of leishmaniasis, facilitating the discovery of novel biomarkers and drugs. Both, amastigotes and promastigotes interfere with various metabolic pathways and exhibit a strong dependence on acquiring and utilizing nutrients from their environment to ensure their survival. Previous research has demonstrated various metabolic factors in *Leishmania* that contribute to its virulence. These parasites target various metabolic pathways as part of their virulence strategy. Both amastigotes and promastigotes rely on intermediates of glucose catabolism and specific amino acids that are essential for their survival and virulence [109]. Recent studies reveal that infected macrophages preferentially upregulate oxidative phosphorylation over glycolysis, driven by LPG. The infection upregulates nuclear and mitochondrial genes linked to mitochondrial biogenesis, including IRG-1 (itaconate synthesis), and doubles the mtDNA/nDNA ratio in an IFNAR-dependent manner. This shift boosts mitochondrial function and energy production, aiding parasite survival [110,111]. In *L. donovani*, thermotolerance is associated with the A2 virulence factor. The A2 protein, specific to the amastigote stage, confers resistance to heat and oxidative stress, enhancing visceral infection when expressed in *L. major*. Studies have shown that A2 colocalizes with BiP, an ER chaperone, suggesting it uses BiP’s functions to manage ER stress in infected macrophages. Therefore, the A2 protein of *Leishmania* may affect macrophage function by helping the parasite survive stress within the macrophage [112,113]. NTPDases hydrolyze nucleoside tri- and di-phosphates to monophosphates, influencing adenosine acquisition, inflammatory responses, immune reactions, and parasitic virulence. In *L. amazonensis*, ATP, ADP, and AMP hydrolysis are significantly higher than in less-virulent species like *L. braziliensis* and *L. major*, with increased virulence linked to higher membrane NTPDase expression [114]. Infections with NTPD1 null mutant amastigotes show no delay in lesion development. NTPD1 affects lipophosphoglycan synthesis, influencing early promastigote infectivity [115]. In *L. infantum chagasi* and *L. amazonensis*, anti-NTPDase-2 antibodies and rNTPDase-2 reduced both adhesion and infection, highlighting the important role of NTPDase in virulence [116].

*Leishmania* growth can be supported by iron derived from heme. A study found that 3 tyrosine residues in the LHR1 transmembrane domain are crucial for heme acquisition and virulence in *L. amazonensis*. Since these unique tyrosine residues are absent in humans, LHR1 could be a potential target for anti-leishmanial drugs [117]. The mitochondrial iron transporter LMIT1 is essential for *Leishmania* mitochondrial function and virulence. It regulates the iron-dependent ROS signals that play a role in the differentiation of *Leishmania* amastigotes [118]. LmABCB3 is an atypical mitochondrial ABC transporter in *Leishmania*, crucial for heme and iron/sulfur cluster biogenesis. Inhibition is lethal, and single-allele deletion impairs amastigote replication and reduces virulence [119]. Also, *Leishmania* LABCG1 and LABCG2 transporters are involved in phosphatidylserine exposure, affecting both virulence and resistance to antimonials [120–122]. Methotrexate reductase (MTHFR) is crucial for folate metabolism and homocysteine regulation. While MTHFR is not essential for *Leishmania* virulence in murine models, its absence may cause mild attenuation. If this attenuation is due to the MTHFR loss, restoring the MTHFR expression should fully recover the parasitic virulence [123]. These findings highlight the potential of targeting metabolic genes to develop new anti-leishmanial therapies.

While comparing virulent and avirulent *L. major* transcriptomes, LmjF.36.3850, a hypothetical protein, was found less expressed in avirulent parasites. Motif analysis showed homology with Snf7 (sucrose non-fermenting protein) and predicted binding to DAG, with an energy value similar to PKCα and PKCβ. Supplementing *L. major*-infected macrophages with OAG (DAG analogue) enhanced PKCα and PKCβ1 phosphorylation, increasing IL-12 and decreasing IL-10. Vaccinated BALB/c mice exhibited higher parasite loads, increased IL-4/IL-10, and unchanged IFN-γ. This suggest LmjF.36.3850 interferes with PKC activation, promoting disease-enhancing T cell subsets and positioning it as a potential virulence factor [124].

A recent study has characterized LdTyrPIP_22, a product of the LDBPK_220120.1 gene, which is identified as the first atypical dual specificity lipid-like P-Tyr and PI phosphatase in *L. donovani*. This group confirms the expression of *LDBPK_220120.1* in *leishmania* promastigotes and axenic amastigotes and characterizes it as a dual-specificity phosphatase capable of dephosphorylating both phosphotyrosine residues and monophosphorylated PI(3)P/PI(4)P *in vitro*. LdTyrPIP_22, a conserved phosphatase across various *Leishmania* species, has been hypothesized to play a role in regulating endocytic and exocytic pathways, as well as in the differentiation from metacyclic promastigotes to amastigotes – key transitions in the parasite’s life cycle. This hypothesis is based on localization studies, which show that LdTyrPIP_22 is detected in the flagellar pocket, a site associated with endocytosis. Also, under conditions mimicking the host environment (37°C, pH 5.5), the protein relocalizes to the flagellar pocket and vesicles, suggesting a possible role in phosphoinositide signaling related to trafficking and differentiation. However, this study provides no direct evidence confirming these functions. Given that phosphoinositides constitute 10% of the total phospholipid content of *Leishmania* promastigotes, their metabolism remains understudied [125]. The *L. mexicana* ortholog of LdTyrPIP_22 (LmxM.22.0250) has been identified as LmDUSP1, a key virulence factor. The LmDUSP1-encoding gene is thought to have been acquired via horizontal gene transfer from bacteria. Interestingly, its orthologues in several bacterial pathogens, including Mycobacterium tuberculosis and Listeria monocytogenes, have been linked to virulence. In *L. mexicana*, targeted ablation of *LmxM.22.0250* resulted in significantly attenuated virulence in primary mouse macrophages; therefore, it is considered to promote *Leishmania* infectivity and persistence in vertebrate hosts [126].

In parallel, another study focused on PX-domain-containing proteins in Kinetoplastea, which includes *Leishmania* and *Trypanosoma* spp. A comprehensive bioinformatics analysis identified 170 PX-proteins, which may function in key phosphoinositide-driven processes such as signal transduction, protein transport, and membrane trafficking. The study classified these proteins into five subfamilies based on structural domain configurations and revealed both evolutionarily conserved and genus-specific architectures [127].

## Coordinated expression of leishmania virulence factors during distinct infection stages

The virulence factors of *Leishmania* are not expressed uniformly but are tightly regulated through a highly coordinated network of molecular events that operate in a stage-specific and context-dependent manner, allowing the parasite to adapt to distinct microenvironments and optimize its survival and infectivity [4]. As *Leishmania* transitions from the sandfly vector to the mammalian host, it undergoes substantial transcriptional and post-translational remodeling in response to changes in temperature, pH, and nutrient availability, leading to the coordinated expression of virulence factors that are precisely tailored to the demands of each microenvironment [128]. During the promastigote stage in the sandfly vector, surface molecules such as LPG, GP63, and KMP-11 are highly expressed, facilitating attachment to the midgut epithelium, immune suppression, and subsequent phagocytosis by host macrophages [129]. Upon entry into the mammalian host and differentiation into amastigotes within macrophage phagolysosomes, the expression profile shifts: these molecules are significantly downregulated, while a distinct set of effectors, including EF-1α, LdTyrPIP_22, cysteine proteases, and LdMAPK12, become dominant, aiding in adaptation to the acidic, oxidative environment of the phagolysosome and promoting immune evasion [130]. These amastigote-enriched factors actively rewire host macrophage signaling, suppress antimicrobial responses, and enhance intracellular survival. The co-regulation of these virulence factors is orchestrated by both parasite-intrinsic mechanisms – such as epigenetic modifications, stage-specific promoters, and post-transcriptional regulation – and host-derived signals, including cytokines and environmental cues (e.g. temperature, pH, and oxidative stress). Several of these virulence factors show co-regulation, influenced not only by intrinsic-stage conversion signals but also by host cytokines (e.g. IL-10, IFN-γ) and TLR cross-talk. For instance, LdMAPK12 expression is suppressed by pro-inflammatory cytokines but enhanced in anti-inflammatory environments [83]. Moreover, *Leishmania* modulates host enzymes such as MTMR6 and SHP-1, which act as proxy virulence factors, and their activity is induced or sustained differentially across life cycle stages [75,131]. This dynamic and coordinated expression landscape highlights the parasite’s ability to temporally synchronize its virulence repertoire with its developmental program and immune microenvironment, ensuring maximal fitness at each phase of infection, which helps maximize its ability to invade, survive, and proliferate within its host.

## Virulence factors as therapeutic targets

Targeting the host virulence factors could potentially lead to the development of novel therapeutic strategies for treating Leishmaniasis. *Leishmania* virulence factors exert a significant impact on host cell signaling pathways, orchestrating a complex interplay that influences the course of leishmaniasis and has important implications for therapeutic strategies. Anti-virulence therapy is a promising new strategy that targets pathogens by depriving them of their virulence factors instead of killing them. Understanding these aspects of *Leishmania* virulence factors and their interactions with host signaling pathways is essential for developing targeted therapeutic strategies and vaccines to combat leishmaniasis. Studies has shown that, drugs designed to restore disrupted signaling pathways, such as those that target host kinases or immune modulators, hold promise for improving treatment outcomes. Combining these interventions with traditional antiparasitic drugs may represent a multifaceted approach to tackle leishmaniasis. Insights into the impact of *Leishmania* virulence factors on host signaling pathways inform vaccine development efforts, potentially leading to effective preventive measures against this challenging disease.

## Conclusion

Virulence factors play crucial roles in *Leishmania* infection and are probable drug targets that lay the basis for rational drug design. More investigation into the virulence factors of various *Leishmania* could advance our understanding of disease pathogenesis and contribute to the development of novel therapeutics. Further research on *Leishmania* genome will help to identify novel virulence factors. Advances in genomics and proteomics can help in the discovery of previously unknown proteins and pathways that contribute to the parasites ability to infect and survive within host cells. The *Leishmania* virulence factors play a decisive role in establishing parasitic infection. Pathogenic factors such as lipophosphoglycan, cysteine proteases, KMP11, GP63, and others are crucial determinants of leishmaniasis by aiding the parasite evasion of host defenses. These virulence factors evade the immune response by altering signaling pathways and modifying chemokine and cytokine profiles, leading to immunosuppression during leishmaniasis. Identifying and characterizing these virulence factors is essential for advancing diagnostic and therapeutic approaches. This review highlights the potential of these proteins as vaccine candidates and underscores the need for continued research to develop novel vaccines and therapeutic targets to effectively combat leishmaniasis.

## Data Availability

Data was not used. We review the information already published from different laboratories, as cited.

## Abbreviations

CL: Cutaneous *Leishmania*sis;
GP63: glycoprotein 63;
iNOS: Induced nitric oxide synthase;
LPG: lipophosphoglycan;
MCL: Mucocutaneous *Leishmania*sis;
NO: nitric oxide;
ROS: reactive oxygen species;
VL: Visceral *Leishmania*sis
